# Prediction of drug-disease associations based on ensemble meta paths and singular value decomposition

**DOI:** 10.1186/s12859-019-2644-5

**Published:** 2019-03-29

**Authors:** Guangsheng Wu, Juan Liu, Xiang Yue

**Affiliations:** 1 School of Computer Science, Wuhan University, Wuhan, 430072 People’s Republic of China; 2 Suzhou Institute of Wuhan University, Suzhou, 215123 People’s Republic of China; 3 Department of Computer Science and Engineering, The Ohio State University, Ohio, 43210 USA

**Keywords:** Drug repositioning, Drug development, Meta path, Commuting matrix, Singular value decomposition

## Abstract

**Background:**

In the field of drug repositioning, it is assumed that similar drugs may treat similar diseases, therefore many existing computational methods need to compute the similarities of drugs and diseases. However, the calculation of similarity depends on the adopted measure and the available features, which may lead that the similarity scores vary dramatically from one to another, and it will not work when facing the incomplete data. Besides, supervised learning based methods usually need both positive and negative samples to train the prediction models, whereas in drug-disease pairs data there are only some verified interactions (positive samples) and a lot of unlabeled pairs. To train the models, many methods simply treat the unlabeled samples as negative ones, which may introduce artificial noises. Herein, we propose a method to predict drug-disease associations without the need of similarity information, and select more likely negative samples.

**Results:**

In the proposed EMP-SVD (Ensemble Meta Paths and Singular Value Decomposition), we introduce five meta paths corresponding to different kinds of interaction data, and for each meta path we generate a commuting matrix. Every matrix is factorized into two low rank matrices by SVD which are used for the latent features of drugs and diseases respectively. The features are combined to represent drug-disease pairs. We build a base classifier via Random Forest for each meta path and five base classifiers are combined as the final ensemble classifier. In order to train out a more reliable prediction model, we select more likely negative ones from unlabeled samples under the assumption that non-associated drug and disease pair have no common interacted proteins. The experiments have shown that the proposed EMP-SVD method outperforms several state-of-the-art approaches. Case studies by literature investigation have found that the proposed EMP-SVD can mine out many drug-disease associations, which implies the practicality of EMP-SVD.

**Conclusions:**

The proposed EMP-SVD can integrate the interaction data among drugs, proteins and diseases, and predict the drug-disease associations without the need of similarity information. At the same time, the strategy of selecting more reliable negative samples will benefit the prediction.

## Background

De novo drug discovery is a complex systematic project which is expensive, time-consuming and with high failure risks. As reported, it will take 0.8–1.5 billion dollars and about 10–17 years to bring a small molecule drug into market, and during the development stage, almost 90% of the small molecules can not pass the Phase I clinical trial and finally be eliminated [1, 2]. For the approved drugs in market, their pharmacological and toxicological properties are clear and the drug safeties are often guaranteed, but only some of their indications are found. For example, there are 2589 approved small molecule drugs in DrugBank [3], and more than 25000 diseases in UMLS medical database [4], resulting in over 60 millions of drug-disease pairs. However, only less than 5% of the drug-disease pairs were identified to have therapeutic relationships, and most of the drug-disease relationships are unknown [5]. Therefore, to discover the new indications of approved drugs, known as drug repositioning, can greatly save money and time, especially can improve the success rate, has become a promising alternative for de novo drug development.

Historically, finding a new indiction of a drug is likely to be an accidental event with a bit of luck. For example, Minoxidil, originally for the treatment of hypertension, was found by chance to have the treatment efficacy for hair loss [6]; Sildenafil (trade name: Viagra), originally for the treatment of angina, was occasionally found to have the potential to treat erectile dysfunction [7]. Such occasional findings of the drugs’ new indictions suggest a new methodology of drug development. However, the “pot-luck” approach can not promise drug repositioning effectively and efficiently. It is necessary to develop a computational method that helps to redirect approved drugs. Fortunately, with the accumulation of multiple omics data and the development of machine learning methods, it is possible to mine the drugs’ potential indications in silico. Up to now, many computational methods have been proposed to find new indictions of drugs by predicting potential treatment relationships of drug-disease pairs.

Based on the hypothesis that the gene expression signature of a particular drug is opposite to the gene expression signature of a disease, some gene expression based methods [8, 9] have been proposed. Noticing that such kind of methods may fail to consider the different roles of genes and their dependencies at the system level, system-level based approach that integrates the gene expressions and related network has recently been proposed [10].

Recently, along with the increase of drugs and diseases related multi-omics data, many methods have been proposed to integrate multiple sources of data to predict the drug-disease interactions based on machine learning techniques. Gottlieb et al. proposed a method (PREDICT) to predict new associations between drugs and diseases by integrating five drug-drug similarities and two disease-disease similarities data [11]. Wang et al. proposed a computational framework based on a three-layer heterogeneous network model (TL-HGBI) by integrating similarities and interactions among diseases, drugs and drug targets [12]. Luo et al. utilized some comprehensive similarities about drugs and diseases, and proposed a Bi-Random walk algorithm (MBiRW) to predict potential drug-disease interactions [13]. Martinez et al. developed a method named DrugNet for drug-disease and disease-drug priorization by integrating heterogeneous data [14]. Wu et al. integrated comprehensive drug-drug and disease-disease similarities from chemical/phenotype layer, gene layer and treatment network layer, and proposed a semi-supervised graph cut method (SSGC) to predict the drug-disease associations [15]. Moghadam et al. adopted the kernel fusion technique to combine different drug features and disease features, and then built SVM models to predict novel drug indications [16]. Liang et al. integrated drug chemical information, target domain information and gene ontology annotation information, and proposed a Laplacian regularized sparse subspace learning method (LRSSL) to predict drug-disease associations [17]. Zhang et al. introduced a linear neighborhood similarity [18] and a network topological similarity [19], then proposed a similarity constrained matrix factorization method (SCMFDD) to predict drug-disease associations by making use of known drug-disease associations, drug features and disease semantic information [20].

However, most of the existed methods are facing two main problems: one is that most of them are based on the hypothesis that similar drugs treat similar diseases, thus they need the similarity information between drugs, proteins, diseases, and so on. However, the similarity data can be not easily obtained. People often need to customize a program to collect data and to calculate the similarities so as to satisfy their own needs. Moreover, the calculation of similarity scores depends on the adopted measures, which may lead that the similarity score of a pair varies dramatically from one method to another. For example, two proteins are similar according to their structures, while they may be dissimilar according to their sequences. Even worse, some features required for calculating the similarities may be unknown or unavailable, resulting that these methods fail to work [21]. The other problem is that supervised learning based methods usually need both positive and negative samples to train the prediction models, whereas the drug-disease pair data, like other biological data, is lack of experimental validated negative samples. To train the models, most of the existing methods randomly select some unlabeled samples as the negative ones. Obviously, such strategy is very rough, for we are not sure whether there are some positive samples uncovered in the unlabeled data.

**Fig. 1:**
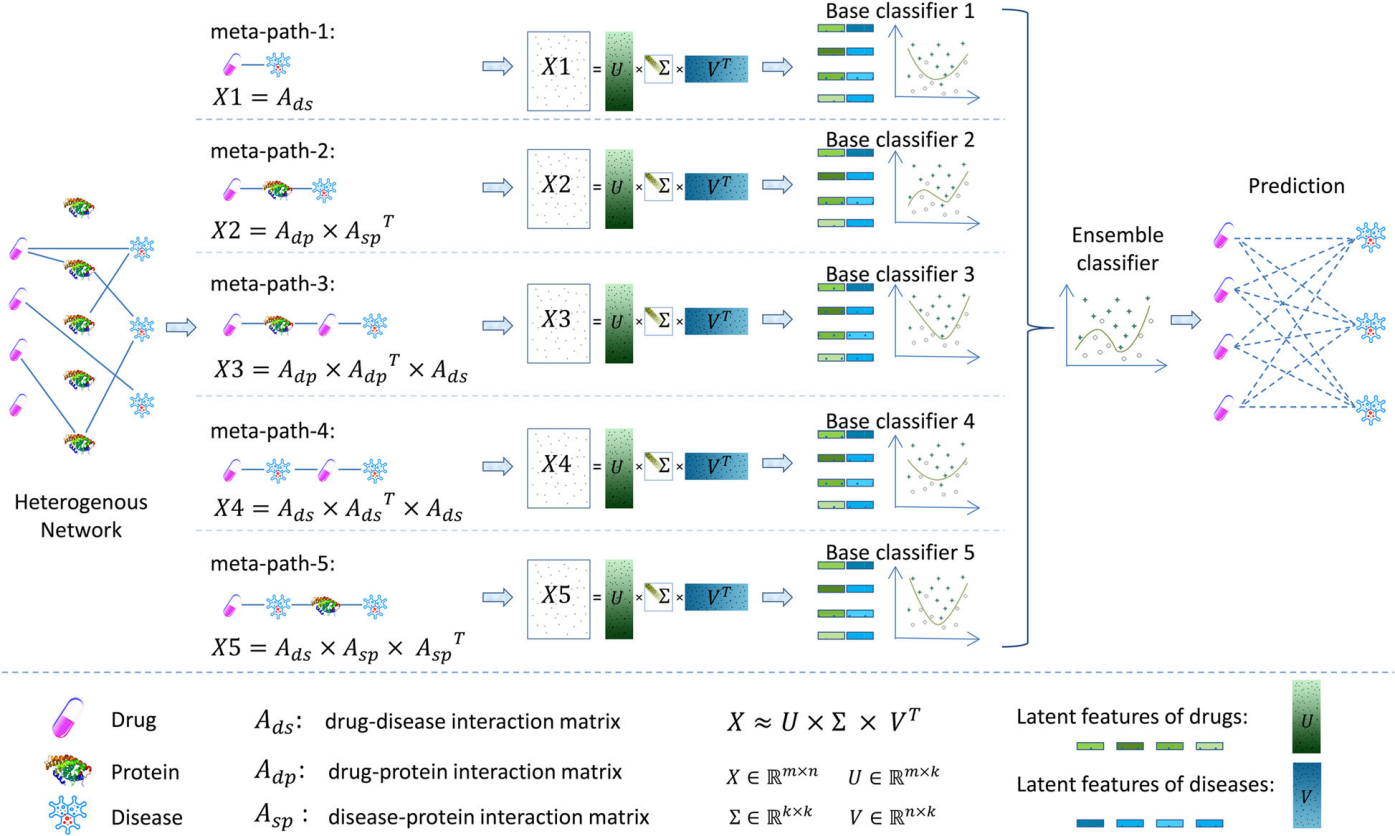
The framework of our proposed EMP-SVD

In this paper, we propose a method, called EMP-SVD (Ensemble Meta Paths and Singular Value Decomposition), to detect drug-disease treatment relations by using drug-disease, drug-protein and disease-protein interaction data. Unlike other methods, EMP-SVD needs no similarity information at all. In order to integrate different kinds of interaction data and consider different dependencies, we introduce five meta paths. For each meta path, we first generate a commuting matrix based on the corresponding interaction data, and then get latent features of drugs and diseases by using SVD (Singular Value Decomposition). All drug-disease pairs can be represented by the features. Finally, we train a base classifier by using the Random Forest algorithm. Five base classifiers are combined as an ensemble model to predict the drug-disease interactions. The framework of our method is shown in Fig. 1. In order to train out a more reliable prediction model, we select more likely negative ones from unlabeled samples under the assumption that non-associated drug and disease pair have no common interacted proteins, which is different from other methods. To evaluate our proposed method, we will compare it with the state-of-the-art methods, and also do case studies by literature investigation.

## Materials and methods

### Data sets

In this paper, we mainly made use of the interaction data of drug-disease, drug-protein and disease-protein to build the prediction model. We collected such data from DrugBank [3, 22, 23], OMIM [24] and Gottlieb’s data set [11]. Concretely, we collected 4642 drug-protein interaction data from DrugBank, involving 1186 drugs and 1147 proteins; 1365 disease-protein interactions from OMIM, involving 449 diseases and 1147 proteins; and 1827 drug-disease interactions from Gottlieb’s data set, involving 302 disease, 551 drugs. Obviously, the heterogenous network composed of drugs, proteins, diseases and the known interactions is sparse. The statistic of the data is shown in Table 1.

**Table 1:** Statistic information of the drug-protein-disease heterogenous network

Type	Property	Number(Density)
Nodes	Drug	1186
	Protein	1147
	Disease	449
Known interactions	Drug ⇔ Protein	4642 (0.0034)
	Disease ⇔ Protein	1365 (0.0027)
	Drug ⇔ Disease	1827 (0.0034)

Density= #known interactions between node1 and node2 / (#node1 * #node2)

Although our method does not need the similarity information, most of other machine learning based methods do need. For the convenience of comparison, we still collected the chemical structure of drugs and the sequence data of proteins from DrugBank. We computed the drug-drug chemical similarities according to their SMILES strings [25] via Openbabel tool [26], and the protein-protein similarities according to the sequence data by Smith-Waterman algorithm [27]. Moreover, we directly downloaded the disease-disease similarities from MimMiner [28].

### Definitions and notations

In this section, we will give the formal definitions and notations used in this paper.

#### **Definition 1**

(Heterogeneous drug-protein-disease network schema). For a given heterogenous drug-protein-disease network *G*=(*V,E*), where *V*=*D*∪*P*∪*S*, *D*, *P* and *S* are the sets of drug, protein, disease nodes in the network respectively, while *E*=*E*_*d,p*_∪*E*_*p,d*_∪*E*_*p,s*_∪*E*_*s,p*_∪*E*_*d,s*_∪*E*_*s,d*_ are the sets of heterogeneous links in *G*, which include the “binds to” link between drugs and proteins, “causes/caused by” link between proteins and diseases, “treats/treated by” link between drugs and diseases. The schema of *G* can be defined as $M_{G} = (\mathcal {T,R})$, where $\mathcal {T}=\{Drug, Protein, Disease\}$, $\mathcal {R}=\{binds~to, cuases, caused~by, treats, treated~by\}$, $\mathcal {T}$ and $\mathcal {R}$ are the sets of node types and link types in *G*, respectively.

**Fig. 2:**
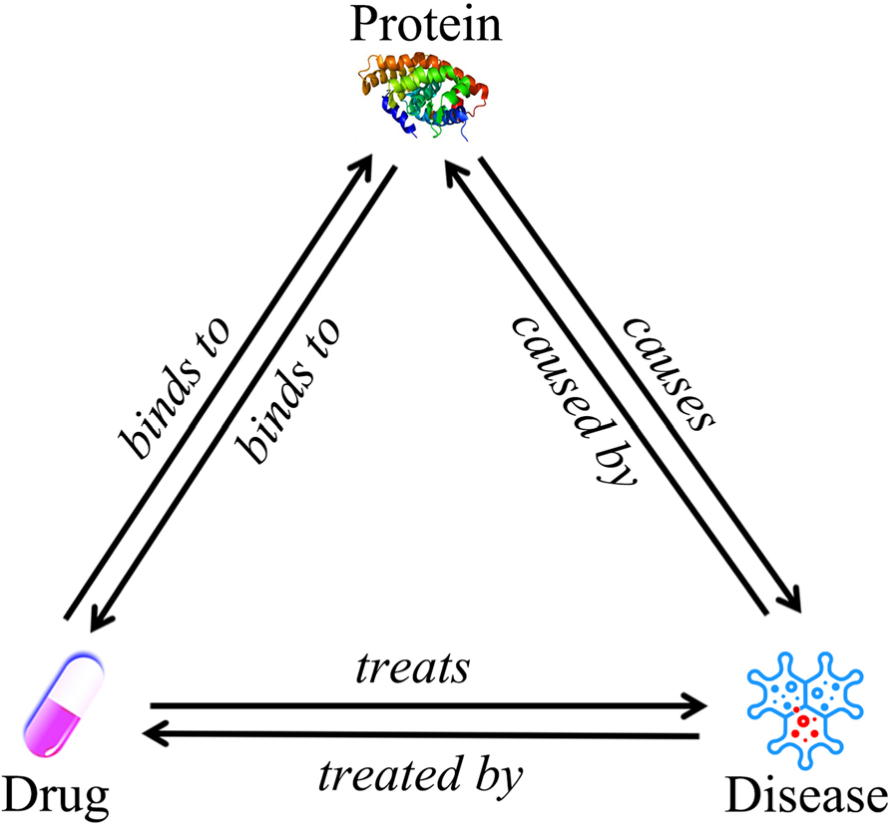
Schema of drug-protein-disease heterogeneous network

The network schema *M*_*G*_ severs as a template of a network *G*. For a drug-protein-disease heterogenous network, the network schema is shown in Fig. 2.

#### **Definition 2**

(Heterogenous network meta path) Based on a given heterogenous network schema $M_{G} = (\mathcal {T,R})$, $\mathcal {P} = T_{1} \xrightarrow {R_{1}} T_{2}\xrightarrow {R_{2}}...\xrightarrow {R_{k-1}} T_{k} $ is defined to be a heterogenous network meta path in network *G*, where $T_{i} \in \mathcal {T}$, *i*∈{1,2,...,*k*} and $R_{i} \in \mathcal {R}$, *i*∈{1,2,...,*k*−1} and if (*T*_1_,*T*_2_,...,*T*_*k*_ are not all the same) ∨ (*R*_1_,*R*_2_,...,*R*_*k*−1_ are not all the same).

For simplicity, we also omit the link types in denoting the meta path if there is no multiple links between the two types, for examples, $\mathcal {P} = T_{1} \xrightarrow {} T_{2}\xrightarrow {}...\xrightarrow {} T_{k} $ denotes the meta path $\mathcal {P} = T_{1} \xrightarrow {R_{1}} T_{2}\xrightarrow {R_{2}}...\xrightarrow {R_{k-1}} T_{k} $. The length of $\mathcal {P}$ is the number of links in $\mathcal {P}$.

#### **Definition 3**

(Commuting matrix [29]) Given a network *G*=(*V*,*E*) and its network schema *M*_*G*_, a commuting matrix for a meta path $\mathcal {P} = T_{1} \xrightarrow {} T_{2}\xrightarrow {}...\xrightarrow {} T_{k} $ is defined as $X = A_{T_{1} T_{2}} A_{T_{2} T_{3}}...A_{T_{k-1} T_{k}}$, where $A_{T_{i} T_{j}}$ is the adjacency (interaction) matrix between type *T*_*i*_ and type *T*_*j*_. *X*(*i*,*j*) represents the number of path instances between object *u*_*i*_∈*T*_1_ and object *v*_*j*_∈*T*_*k*_ under meta path $\mathcal {P}$.

Since we want to detect the interactions between the drugs and the diseases, we only consider the cases of *T*_1_=*Drug* and *T*_*k*_=*Disease*.

Now that there are only three kinds of nodes (drug, protein and disease) in the heterogenous network, we think the meta path with length greater than three may be too long to contribute to the prediction. Sun’s work also has shown that short meta paths are good enough, and long meta paths may even reduce the quality [29]. Therefore, in this work, we only selected meta paths with length no longer than three. As a result, we select five meta paths described below.

Let *A*_*ds*_ be the drug-disease interaction matrix, *A*_*dp*_ be the drug-protein interaction matrix, and *A*_*sp*_ be the disease-protein interaction matrix, we can get the commuting matrices of the five meta paths as follows:

**Meta-path-1**: Drug $\xrightarrow {{treats}}$ Disease. The commuting matrix of it, denoted as *X*1, can be obtained by:

1 $$ X1 = A_{ds}  $$

**Meta-path-2**: Drug $\xrightarrow {{binds\ to}}$ Protein $\xrightarrow {{causes}}$ Disease. The commuting matrix of it, denoted as *X*2, can be obtained by :

2 $$ X2 = A_{dp} \times A_{sp}^{T}  $$

By using meta-path-2, we can integrate the drug-protein interaction information and the disease-protein interaction information, that is to say, we easily take the protein related information into account.

**Meta-path-3**: Drug $\xrightarrow {{binds\ to}}$ Protein $\xrightarrow {{binds\ to}}$ Drug $\xrightarrow {treats}$ Disease. The commuting matrix of it, denoted as *X*3, can be obtained by:

3 $$ X3 = A_{dp} \times A_{dp}^{T} \times A_{ds}  $$

By using meta-path-3, we can integrate drug-protein interaction and drug-disease interaction information. What’s more, meta-path-3 also indicates that if two drugs share some common proteins, they may have similar indications.

**Meta-path-4**: Drug $\xrightarrow {{treats}}$ Disease $\xrightarrow {{treated\ by}}$ Drug $\xrightarrow {{treats}}$ Disease. The commuting matrix of it, denoted as *X*4, can be obtained by :

4 $$ X4 = A_{ds} \times A_{ds}^{T} \times A_{ds}  $$

By using meta-path-4, we can integrate the drug-disease interaction information. Besides, meta-path-4 also indicates that if two drugs share some common indications, then the indication of one drug may also be the potential indication of another drug.

**Meta-path-5**: Drug $\xrightarrow {treats}$ Disease $\xrightarrow {caused\ by}$ Protein $\xrightarrow {causes}$ Disease. The commuting matrix of it, denoted as *X*5, can be obtained by :

5 $$ X5 = A_{ds} \times A_{sp} \times A_{sp}^{T}  $$

By using meta-path-5, we can integrate the drug-disease interaction and the disease-protein interaction information. What’s more, meta-path-5 also indicates that if two disease share some common proteins, the drug for treating one disease may also be the potential therapeutical drug for another disease.

**Fig. 3:**
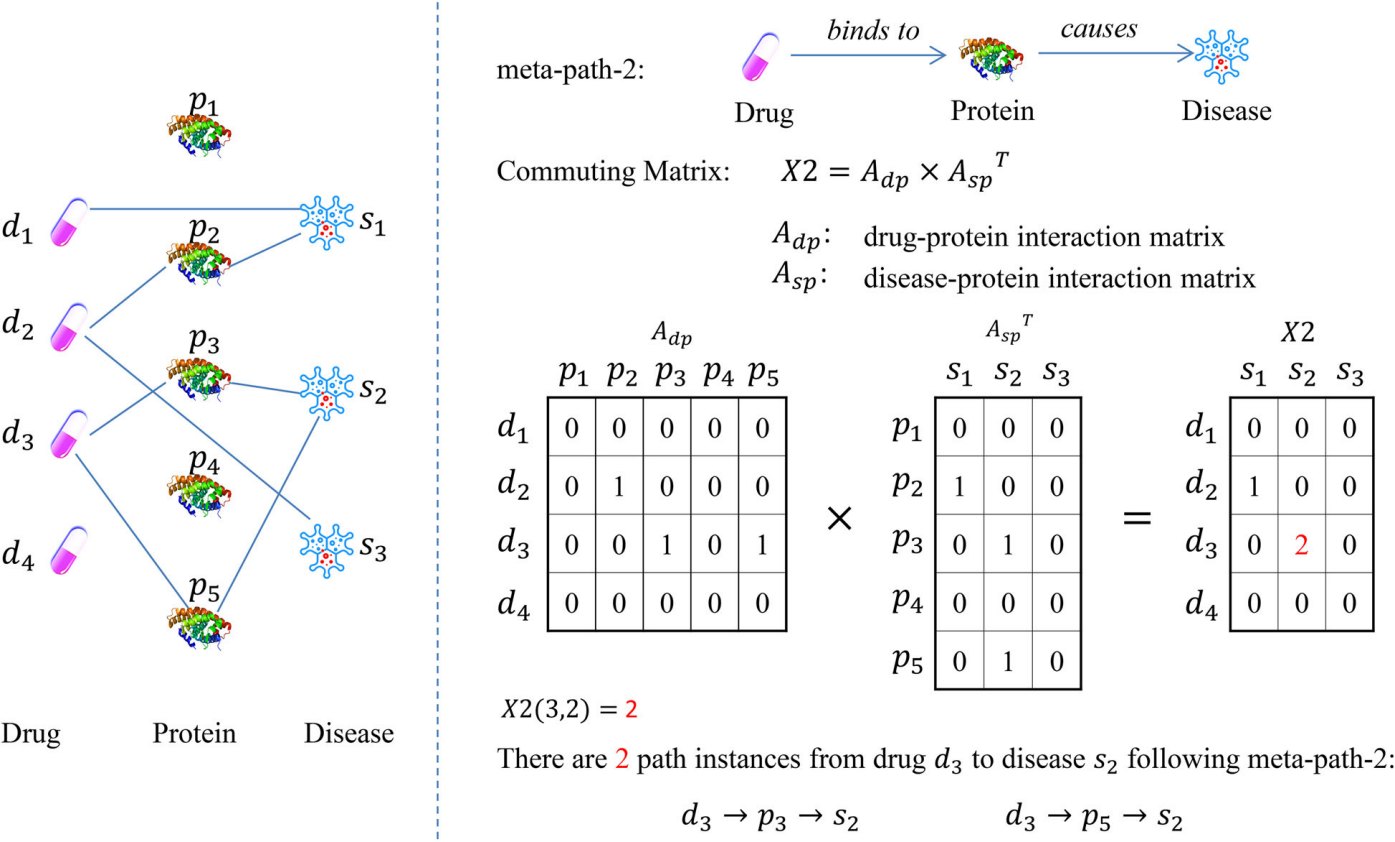
An example of the meaning of commuting matrix

As the definition, the element *X*(*i,j*) of the commuting matrix *X* denotes the number of path instances from drug *d*_*i*_ to disease *s*_*j*_ under the corresponding meta path. We show an example in Fig. 3. There are two path instances from drug *d*_3_ to disease *s*_2_ under **Meta-path-2**, $d_{3} \xrightarrow {} p_{3} \xrightarrow {} s_{2}$ and $d_{3} \xrightarrow {} p_{5} \xrightarrow {} s_{2}$, thus we have *X*2(3,2)=2 in commuting matrix *X*2.

### Feature extraction with singular value decomposition

Now that element *X*(*i,j*) in a commuting matrix *X* denotes the number of path instances from the drug *d*_*i*_ to disease *s*_*j*_, then row *i* in the commuting matrix can be used as features of drug *d*_*i*_, and column *j* can be used as features of disease *s*_*j*_. And we can use the concatenation of them to represent the drug-disease pair. Suppose there are *m* drugs and *n* diseases, we will have *m*+*n* (In this work, *m*=1186,*n*=449) features to represent the drug-disease pair. By contrast, the number of drug-disease pairs is small (We only have 1827 known interactions in this work). Obviously, the feature dimension is relatively high, which is not proper to construct a robust prediction model. Now that the singular value decomposition (SVD) has been successfully used to reduce the dimension in many researches, we also employed SVD to extract small number of features in our work.

By using SVD, the commuting matrix $X \in \mathbb {R}^{m \times n}$ can be factorized into *U*, *Σ* and *V* such that

6 $$ X = U \Sigma V^{T}  $$

where $U \in \mathbb {R}^{m \times m}$, $\Sigma \in \mathbb {R}^{m \times n}$ and $V \in \mathbb {R}^{n \times n}$. The diagonal entries of *Σ* are equal to the singular values of *X* (Other elements in *Σ* other than diagonal entries are 0). The columns of *U* and *V* are, respectively, left- and right- singular vectors for the corresponding singular values.

As is known to all, the magnitude of the singular values represents the importance of the corresponding vectors; and in *Σ*, the singular values are ordered in descending order. Moreover, in most cases, the sum of the first 10% or even 1% of the singular values is over 99% of the total sum of all singular values. Specifically in this drug-disease associations prediction problem, in the biomedical meaning, the most useful information about drug and disease features will be included in the first 10% even less singular values. In the process of dimensionality reduction, the useful data will not be lost, but the redundant information will be discarded. That is to say, we can use the top *r* singular values to approximate the matrix *X*:

7 $$  X \approx U_{m \times r} \Sigma_{r \times r} {V^{T}}_{r \times n}  $$

where *r*≪*min*(*m,n*).

Row *i* in *U* can be used as latent features of drug *d*_*i*_, and row *j* in *V* can be used as latent features of disease *s*_*j*_. As a result, the dimension of the latent feature vector of each drug-disease pair can be reduced to 2∗*r*. In this work, we will introduce a parameter *latent_feature_percent* far less than 1 (say 1%, 2%,...) to control the value of *r* such that *r*=*latent_feature_percent*×*min*(*m,n*).

### Selection of likely negative samples from unlabeled drug-disease pairs

To build a prediction model by using supervised learning, we need both positive and negative samples. The known drug-disease treatment relations are positive samples. Being lack of validated negative samples, most methods simply select some of unlabeled samples as negative ones by random. However, the unlabeled samples are not necessarily negative, some of them may be positive samples that still remain uncovered by experiments [30]. Different with other methods, we try to find more reliable negative samples from the unlabeled ones in this work.

If a drug shares some proteins with a disease, then the drug may have potential to treat the disease. Intuitively, if a drug and a disease have no common related proteins, we can think the disease is not the indication of the drug, and thus the drug-disease pair is more likely a negative sample. By this means, we can select out more reliable negative samples from the unlabeled pairs based on the drug-protein and disease-protein interactions information. The procedure is listed in Algorithm 1.


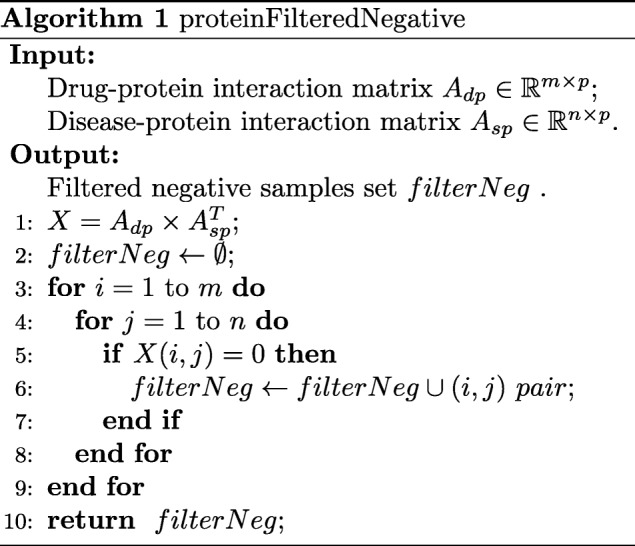


### Construction and ensemble of classifiers

The five meta paths we have selected to integrate heterogeneous data reflect different aspects of the drug-disease treatment relationship, such as two drugs with common proteins having similar indications, two drugs sharing one common indication also sharing another indication, and so on. Thus we can build five base classifiers for the prediction of drug-disease treatment relations from different sides. In our work, the base classifiers are built based on the Random Forest algorithm which was implemented by using the *RandomForestClassifier* function in the scikit-learn package [31], we set the number of trees as 256.

Since ensemble learning can often help to improve the performances [32, 33], after the five base classifiers are constructed, we can obtain an ensemble classifier. For an input of drug-disease pair, each base classifier outputs two probabilities indicating that the pair being negative and positive respectively. Since we want to know whether the pair has treatment relation, we only take the positive probability as considered in the ensemble model.

For a drug-disease pair *x* with unknown label, suppose the predicted score (probability) of each base classifier be *h*_*i*_(*x*),*i*=1,2,...5, we used average strategy to get the final score of the ensemble model:

8 $$ H(x) = \frac{1}{5} \sum\limits_{i=1}^{5} h_{i}(x)  $$

If *H*(*x*) is greater than a predetermined threshold, then the sample *x* is predicted as the positive. Because *F*_1_-measure is a comprehensive metric, in this work, we let the program automatically determine the threshold value when *F*_1_-measure reaches the maximum value, which is the same strategy as the other researchers used.

## Experiments and results

We perform 5-fold cross validation to evaluate our method. Since the filtered negative samples are more than the positive ones, we randomly select a subset from them that with size equal to the positives, and use the balanced data to train the models. We first select the appropriate number of features according to the relationship of the model performance and the feature number. Then we did three kinds of evaluation experiments: (1) We investigate whether our negative samples filtering strategy can help to improve the prediction performance; (2) We compare EMP-SVD with other state-of-the-art methods by using the same data; (3) We check the practicality of our method by doing case studies.

### Evaluation metrics

Just as most other work, we performed 5-fold cross validation in the experiments. To evaluate performance of a method, there are some common metrics: Precison (*PRE*), Recall (*REC*), Accuracy (*ACC*), Matthews Correlation Coefficient (*MCC*) and *F*_1_-measure (*F*_1_). They can be calculated according to the following equations:

9 $$\begin{array}{*{20}l} PRE &= \frac{TP}{TP+FP} \end{array} $$

10 $$\begin{array}{*{20}l} REC &= \frac{TP}{TP+FN} \end{array} $$

11 $$\begin{array}{*{20}l} ACC &= \frac{TP+TN}{TP+FP+TN+FN} \end{array} $$

12 $$\begin{array}{*{20}l} MCC &= \frac{TP \times TN - FP \times FN}{ \sqrt{ (TP+FP)(TP+FN)(TN+FP)(TN+FN) }} \end{array} $$

13 $$\begin{array}{*{20}l} F_{1} &= \frac{2 \times PRE \times REC}{PRE+REC} \end{array} $$

where *TP*, *FP*, *TN* and *FN* denote the number of true positive samples, false positive samples, true negative samples and false negative samples, respectively.

Since Precision(*PRE*) and Recall(*REC*) have some conflicts, in general, a classifier gets a higher *PRE* will have a lower *REC*, and vise versa. To get a comprehensive performance, Area Under Precison-Recall Curve(*AUPR*) and Area Under Receiver Operating Characteristic Curve(*AUC*) are often used. *AUPR* takes both *PRE* and *REC* into account, *AUC* takes both the true positive rate(*TPR*, the same as *REC*) and the false positive rate (*FPR*) into account, so they are comprehensive metrics. At the same time, with the help of the curves we can intuitively find which classifier is better. Therefore, in this work, we adopted *AUPR* and *AUC* as the main metrics.

### Determination of appropriate number of features

Parameters are often used in existing computational methods, which limits the generalization of a model. So, it will be better to use fewer parameters or to get an analytical solution.

**Fig. 4:**
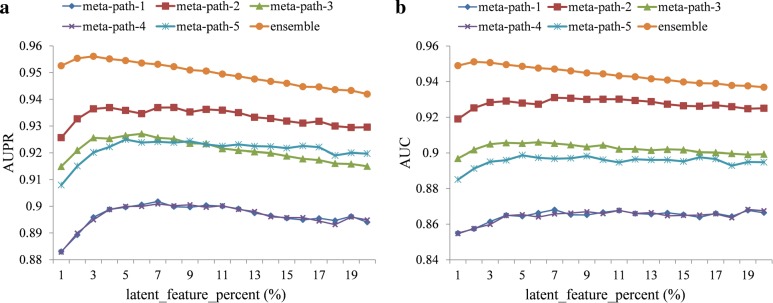
Influence of different *latent_feature_percent* on the **a** AUPR **b** AUC

In this work, we just need to determine the number of singular values (corresponding to the feature number that is controlled by the parameter *latent_feature_percent*) during the model construction, which is very different with most state-of-the-art methods. Just mentioned above *r*≪*min*(*m,n*), so we set *latent_feature_percent* as 1%, 2%, 3%,......, 20% respectively, and the performance curves of five base classifiers and the ensemble one with different *latent_feature_percent* are shown in Fig. 4. The results have shown that the performances of the ensemble classifier are better than other five base classifiers, illustrating that our ensemble rule is effective. Moreover, the performances of the six classifiers are robust across different parameter settings. Anyway, we set *latent_feature_percent* as 3% according to the curves in this work.

We also find that the performances of classifiers based on meta-path-1 and meta-path-4 are the worst. Noticing that both meta-path-1 and meta-path-4 just take drug-disease interactions into consideration, while the other three meta paths contain more information on drug-protein or protein-disease interactions, we think integrating more interaction information into the meta path can help to improve the performance of the classifier.

### Investigation of the filtering strategy of negative samples

Being lack of validated negative samples, most of the other methods randomly select unlabeled samples to be negative ones. However, the unlabeled samples are not necessarily negative, some of them may be positive samples still uncovered by experiments. So in this work we selected out more likely negative samples from unlabeled ones according to the common protein information (as described in Algorithm 1). As shown in Table 2, all the classifiers achieve better performances in most metrics when using our negative samples filtering strategy. We also noted that the improvement is little, which may due to the fact that the known drug-protein interactions and disease-protein interactions are too few (with density of 0.0034 and 0.0027, as shown in Table 1), resulting that very few proteins could be used in the filtering process. Anyway, our strategy for selecting more reliable negative samples is useful, feasible and interpretable. We believe that along with the increase of interactions data, we will get more reliable negative samples and thus achieve more great performance improvements.

**Table 2:** Performances comparison with different negative samples selecting strategies (random strategy is denoted “random”, our strategy is “reliable”)

Methods	AUPR		AUC		PRE		REC		ACC		MCC		F1	
	Random	Reliable	Random	Reliable	Random	Reliable	Random	Reliable	Random	Reliable	Random	Reliable	Random	Reliable
meta-path-1	0.894	0.896	0.859	0.861	0.786	0.771	0.875	0.891	0.835	0.835	0.673	0.677	0.827	0.826
meta-path-2	0.930	0.936	0.925	0.928	0.873	0.850	0.839	0.873	0.850	0.861	0.702	0.722	0.855	0.860
meta-path-3	0.921	0.926	0.902	0.905	0.826	0.832	0.862	0.883	0.843	0.858	0.690	0.719	0.842	0.855
meta-path-4	0.894	0.895	0.858	0.860	0.782	0.790	0.882	0.867	0.836	0.832	0.676	0.667	0.828	0.826
meta-path-5	0.918	0.920	0.892	0.895	0.809	0.800	0.900	0.925	0.859	0.865	0.721	0.737	0.852	0.858
ensemble	0.954	0.956	0.949	0.951	0.924	0.913	0.837	0.854	0.871	0.876	0.745	0.755	0.878	0.882

### Comparison with other methods

In this section, we compare EMP-SVD with state-of-the-art methods to demonstrate the superior performance of our method. PREDICT [11] and TL-HGBI method [12] are classical methods used to predict the drug-target and drug-disease interactions. MBiRW [13], LRSSL [17] and SCMFDD [20] are the methods proposed in these two years, and achieved high performance in the prediction of drug-disease interaction. So we choose these state-of-the-art methods to compare.

PREDICT calculates the score of a given drug-disease pair (*d*_*r*_,*d*_*i*_) according to all the known drug-disease pairs $\left (d_{r}^{\prime },d_{i}^{\prime }\right)$ associated with that given pair by equation $Score(d_{r},d_{i})= {\underset {d_{r}^{\prime },d_{i}^{\prime } \neq d_{r},d_{i}}{\max }} \sqrt { S \left (d_{r},d_{r}^{\prime }\right) \times S \left (d_{i},d_{i}^{\prime }\right) }$, where $S \left (d_{r},d_{r}^{\prime }\right)$ is drug-drug similarity and $S\left (d_{i},d_{i}^{\prime }\right)$ is disease-disease similarity. TL-HGBI is a three layer heterogenous network model, which makes use of the similarities and interactions of drugs, diseases and targets by iterative update. MBiRW adjusts the similarities of drugs and diseases by correlation analysis and known drug-disease associations, then uses Bi-random walk algorithm to predict the potential drug-disease associations. LRSSL is a Laplacian regularized sparse subspace learning method used to predict the drug-disease associations which integrates drug chemical information, drug target domain information and target annotation information. SCMFDD is a similarity constrained matrix factorization method for the prediction of drug-disease associations by using known drug-disease interactions, drug features and disease semantic information.

We obtained the source code of PREDICT, TL-HGBI and SCMFDD from the authors, the code of MBiRW, LRSSL are publicly available, and the parameters were set according to their papers. The parameter *latent_feature_percent* in EMP-SVD was set 3%. To be fair, the five parts data were kept the same division in all methods when conducting 5-fold cross validation.

**Fig. 5:**
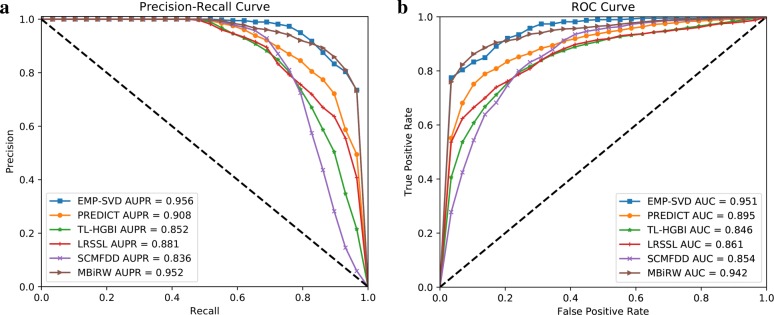
**a** Precision-Recall Curve **b** ROC Curve of EMP-SVD and compared methods

As shown in Table 3, compared with other five state-of-the-art methods which make use of several kinds of similarities as well as the interaction data, the proposed classifier EMP-SVD only uses the known interaction data but achieves better performances in most metrics, especially the comprehensive metrics (AUPR and AUC). To make it more intuitively, we plotted the Precison-Recall Curve and ROC curve, which are shown in Fig. 5a and b, respectively. The AUPR and AUC of the proposed EMP-SVD are 0.956 and 0.951, respectively, better than the compared methods. Hence, it shows the simplicity and effectiveness of our method.

**Table 3:** Performances of proposed EMP-SVD and state-of-the-art methods

Methods	AUPR	AUC	PRE	REC	ACC	MCC	*F*_1_
EMP-SVD	0.956	0.951	0.913	0.854	0.876	0.755	0.882
PREDICT	0.908	0.895	0.809	0.850	0.830	0.662	0.828
TL-HGBI	0.852	0.846	0.829	0.750	0.774	0.552	0.787
LRSSL	0.881	0.861	0.864	0.732	0.770	0.553	0.790
SCMFDD	0.836	0.854	0.926	0.713	0.774	0.575	0.805
MBiRW	0.952	0.942	0.867	0.901	0.884	0.769	0.884

### Case studies

Here, we test the practicality of EMP-SVD for predicting unknown associations. Except for training set composing of the known 1827 drug-disease associations and randomly selected 1827 negative samples by using our strategy, we used the trained EMP-SVD model to predict the associations for other unknown drug-disease pairs, and validate the results by literature investigation.

The new predicted top 20 drug-disease associations are shown in Table 4. We checked them carefully by literature validation and found that 13 of the top 20 predicted associations have been reported in the literatures. And these predicted associations were not originally in our data set, but we could find it out by our method, thus showing the practicality of our proposed EMP-SVD.

**Table 4:** The predicted drug-disease associations (Top 20)

Rank	Score	DrugBank ID	Drug name	OMIM ID	Disease name	Literature validation
1	0.994	DB00776	Oxcarbazepine	239350	Hyperphosphatemia, Polyuria, And Seizures	[38]
2	0.992	DB01234	Dexamethasone	151590	Lichen Sclerosus Et Atrophicus; Lsa	[39]
3	0.991	DB00443	Betamethasone	233810	Growth Retardation, Small And Puffy Hands And Feet, And Eczema	[36]
4	0.991	DB00694	Daunorubicin	236000	Hodgkin Lymphoma	[40, 41]
5	0.987	DB01234	Dexamethasone	146850	Immune Suppression; Is	[42]
6	0.986	DB01013	Clobetasol propionate	233810	Growth Retardation, Small And Puffy Hands And Feet, And Eczema	N.A.
7	0.986	DB00620	Triamcinolone	125600	Dermatosis Papulosa Nigra	N.A.
8	0.986	DB00863	Ranitidine	600263	Helicobacter Pylori Infection, Susceptibility To	[43]
9	0.985	DB00620	Triamcinolone	233810	Growth Retardation, Small And Puffy Hands And Feet, And Eczema	[34, 35]
10	0.984	DB00694	Daunorubicin	267730	Reticulum Cell Sarcoma	[44]
11	0.984	DB00694	Daunorubicin	109543	Leukemia, Chronic Lymphocytic, Susceptibility To, 2	N.A.
12	0.984	DB00773	Etoposide	247640	Lymphoblastic Leukemia, Acute, With Lymphomatous Features; Lall	[45, 46]
13	0.984	DB00214	Torasemide	256370	Nephrotic Syndrome, Early-Onset, With Diffuse Mesangial Sclerosis	N.A.
14	0.983	DB00443	Betamethasone	188030	Thrombocytopenic Purpura, Autoimmune; Aitp	[47]
15	0.981	DB00444	Teniposide	601626	Leukemia, Acute Myeloid; Aml	[48, 49]
16	0.981	DB00481	Raloxifene	215470	Chorioretinal Dystrophy, Spinocerebellar Ataxia, And Hypogonadotropic	N.A.
17	0.980	DB00335	Atenolol	608622	Hypertension, Diastolic, Resistance To	[50]
18	0.980	DB00612	Bisoprolol	608622	Hypertension, Diastolic, Resistance To	[51]
19	0.980	DB00443	Betamethasone	146850	Immune Suppression; Is	N.A.
20	0.980	DB01177	Idarubicin	109543	Leukemia, Chronic Lymphocytic, Susceptibility To, 2	N.A.

N.A.: We haven’t found the literature evidence

It should be noted that Triamcinolone (DrugBank ID: DB00620) and Betamethasone (DrugBank ID: DB00443), as glucocorticoid, are commonly used in the treatment of various skin diseases such as “Eczema” [34–36], and we find that their predicted associations include the disease “Growth Retardation, Small And Puffy Hands And Feet, And Eczema” (OMIM ID:233810). During the process of literature validation, we also find a case of growth retardation and Cushing’s syndrome due to excessive application of betamethasone-17-valerate ointment [37]. In a responsible attitude, we think that whether they can be used to treat the disease “Growth Retardation, Small And Puffy Hands And Feet, And Eczema”, or the usage and dosage should be further carefully studied by the chemists and doctors, especially should be with caution when used on children and pregnant women.

In more details, we checked the predicted potential indications of drug “Amitriptyline” (DrugBank ID: DB00321). Amitriptyline is a tricyclic antidepressant which is often used to treat symptoms of depression with the brand name: Vanatrip, Elavil, Endep. As shown in Table 5, we can find literature evidences to support 8 diseases in the top 10 predictions for Amitriptyline.

**Table 5:** Top 10 predictions for the drug “Amitriptyline”

Rank	Score	OMIM ID	Disease name	Literature validation
1	0.880	102300	Restless Legs Syndrome, Susceptibility To, 1; Rls1	[52]
2	0.877	200170	Acanthosis Nigricans With Muscle Cramps And Acral Enlargement	N.A.
3	0.843	143465	Attention Deficit-Hyperactivity Disorder; Adhd	[53]
4	0.837	600631	Enuresis, Nocturnal, 1; Enur1	[54]
5	0.837	600808	Enuresis, Nocturnal, 2; Enur2	[54]
6	0.817	608088	Neuropathy, Hereditary Sensory And Autonomic, Type I, With Cough And Gastroesophageal Reflux	[55]
7	0.803	145590	Hyperthermia, Cutaneous, With Headaches And Nausea	[56]
8	0.774	164230	Obsessive-Compulsive Disorder; Ocd	N.A.
9	0.769	167870	Panic Disorder 1; Pand1	[57]
10	0.745	600082	Prostatic Hyperplasia, Benign; Bph	[58]

N.A.: We haven’t found the literature evidence

Breast cancer is a relatively common malignant tumor for female, which seriously endangers women’s health and life safety. To discover the potential drugs is of great value. So we also checked the drug list that have been predicted to treat the disease “Breast Cancer” (OMIM ID: 114480). In the top 10 drugs, as shown in Table 6, we found that 8 have been reported to be used in the clinical treatment.

**Table 6:** Top 10 predictions for the disease “Breast Cancer”

Rank	Score	DrugBank ID	Drug name	Literature validation
1	0.931	DB00541	Vincristine	[59–61]
2	0.924	DB00399	Zoledronate	[62]
3	0.902	DB00987	Cytarabine	[63]
4	0.901	DB00884	Risedronate	[64]
5	0.893	DB01073	Fludarabine	N.A.
6	0.889	DB00755	Tretinoin	[65]
7	0.884	DB00762	Irinotecan	[66]
8	0.884	DB00630	Alendronate	[67]
9	0.880	DB01005	Hydroxyurea	[68]
10	0.878	DB01196	Estramustine	N.A.

N.A.: We haven’t found the literature evidence

Therefore, the case studies have further shown the practicality of the proposed method EMP-SVD.

## Conclusions and discussions

To uncover the potential drug-disease associations is an important step in drug development, but it is time-consuming and costly to uncover them by wet experiments. Along with the accumulation of drug and disease related multi-omics data, as well as the development of machine learning techniques, more and more computational methods have been proposed to predict the potential drug-disease associations. To help the prediction, many methods integrate multiple source of data, including drugs, diseases, targets, side effects, and so on. They achieved good performances and could provide a helpful reference to the drug development. Most of them need the similarities of drug and disease related data. However, the similarity data can not be easily obtained, and people often need to customize a program to crawl data and to compute the similarities to satisfy their own need. Even worse, some features needed to calculate the similarity are unknown or unavailable. These methods will not work facing the incomplete data. Besides, being lack of validated negative samples in the prediction of drug-disease associations, most of the machine learning based methods assume the unlabeled samples to be negative ones in the training of the model. Such strategy may input errors because there may be positive samples uncovered in the unlabeled samples. What’s more, most of the existing methods use many parameters in the data integration and the model construction. The parameters are difficult to tune, which limits the generalization ability of the method.

In this work, we proposed a method named EMP-SVD to predict drug-disease interactions based on ensemble meta paths and singular value decomposition. Five meta paths from source node (drug) to end node (disease) were selected to integrate the interaction information of drugs, proteins and diseases. Then the commuting matrices of these meta paths were calculated out, each element indicates the number of path instances between the corresponding drug and disease pair. By using singular value decomposition on the commuting matrices, we can extract small number of latent features of drugs and diseases. In order to get reliable negative samples, we selected those unlabeled samples as negative under the assumption that if a drug and a disease have no common proteins, then there is smaller probability for them to be treatment relationship. Based on each meta path we first built a base classifier, and then combined them to get an ensemble classifier. The experiments results have shown that our proposed EMP-SVD method outperformed several state-of-the-art methods. Better than other methods, EMP-SVD has few parameters and very easy to set. Further more, case studies have shown the predicted new associations could be useful for further biomedical research, which demonstrate the practicality of our method.

Although there are meta path based methods in social network and some other networks, to the best of our knowledge, it is the first work in the prediction of drug-disease associations by using ensemble meta paths and singular value decomposition. Different with many existing methods, we do not need the similarity data which are not easily obtained or sometimes unavailable or unknown. Instead, we just use the interaction data which can be easily accessed in many databases to build the prediction model. The other advantage of method is that there is only one parameter that can easily set. Though we use ensemble strategy to improve the performance, each of the five base classifiers can independently act as the model as well to predict the drug-disease interactions. Since there are many computational methods to predict the target proteins for a new drug such as docking methods. For a new drug which has no known interactions with any diseases, we still can predict its interacted diseases by building classifier using meta-path-2 by making use of drug-protein and protein-disease interactions.

Though the results of our methods are promising, there are still some limitations. Firstly, we only use the information of drugs, proteins and diseases, there are many other information could also be integrated in the further work, such as the information of side effects, pathways, tissues, and so on. Secondly, we only make use of common proteins to select out the negative samples, some other information such as gene expression data can also be used for this purpose. Or we can directly build the model by positive and unlabeled samples based learning method. We will address these issues in the future study.

## Abbreviations

ACC: Accuracy
AUC: Area under ROC curve
AUPR: Area under Precison-Recall curve
FN: False negative
FP: False positive
FPR: False positive rate
MCC: Matthews correlation coefficient
OMIM: Online Mendelian inheritance in man
PRE: Precision
REC: Recall
ROC: Receiver operating characteristic
SMILES: Simplified molecular input line entry specification
SVD: Singular value decomposition
SVM: Support vector machine
TN: True negative
TP: True positive
TPR: True positive rate
UMLS: Unified medical language system
